# Ghrelin receptor antagonist JMV2959 blunts cocaine and oxycodone drug-seeking, but not self-administration, in male rats

**DOI:** 10.3389/fphar.2023.1268366

**Published:** 2023-09-19

**Authors:** Christina R. Merritt, Erik J. Garcia, Victoria D. Brehm, Robert G. Fox, F. Gerard Moeller, Noelle C. Anastasio, Kathryn A. Cunningham

**Affiliations:** ^1^ Center for Addiction Sciences and Therapeutics and Department of Pharmacology and Toxicology, John Sealy School of Medicine, University of Texas Medical Branch, Galveston, TX, United States; ^2^ C. Kenneth and Dianne Wright Center for Clinical and Translational Research, Departments of Psychiatry and Pharmacology and Toxicology, Virginia Commonwealth University School of Medicine, Richmond, VA, United States

**Keywords:** cocaine, oxycodone, growth hormone secretagogue receptor 1α (GHS1αR), Sprague-Dawley rat, self-administration, cue-reinforced drug seeking

## Abstract

The drug overdose crisis has spawned serious health consequences, including the increased incidence of substance use disorders (SUDs), conditions manifested by escalating medical and psychological impairments. While medication management is a key adjunct in SUD treatment, this crisis has crystallized the need to develop additional therapeutics to facilitate extended recovery from SUDs. The “hunger hormone” ghrelin acts by binding to the growth hormone secretagogue receptor 1α (GHS1αR) to control homeostatic and hedonic aspects of food intake and has been implicated in the mechanisms underlying SUDs. Preclinical studies indicate that GHS1αR antagonists and inverse agonists suppress reward-related signaling associated with cocaine and opioids. In the present study, we found that the GHS1αR antagonist JMV2959 was efficacious to suppress both cue-reinforced cocaine and oxycodone drug-seeking, but not cocaine or oxycodone self-administration in male Sprague-Dawley rats. These data suggest a role of the ghrelin-GHS1αR axis in mediating overlapping reward-related aspects of cocaine and oxycodone and premises the possibility that a GHS1αR antagonist may be a valuable therapeutic strategy for relapse vulnerability in SUDs.

## Introduction

Drug overdose deaths in the United States have reached record highs (Ahmad et al., 2023). The explosive increase in deaths is related to licit and illicit opioids while deaths involving cocaine and other psychostimulants also soared dramatically (Ahmad et al., 2023). This crisis spawned additional health consequences, including an increasing incidence of substance use disorders (SUDs), conditions manifested by escalating physical and psychological impairments (SAMHSA, 2021). Psychosocial interventions in combination with medications for opioid use disorder (OUD) reduce mortality, opioid withdrawal, illicit intake, and opioid-seeking in OUD patients, thus substantially improving the odds of successful recovery (Marsden et al., 2019). However, barriers persist in accessing these opioid agonist medications (buprenorphine, methadone) (Sharma et al., 2017), while there are no FDA-approved medications for cocaine use disorder (CUD). Thus, there is a need to pursue creative solutions that promote CUD and OUD recovery.

Preclinical models demonstrated that laboratory animals self-administer psychoactive drugs misused by humans. These models are also useful to study stimuli associated with drug use which contribute to relapse vulnerability during abstinence (Meil and See, 1996; Epstein et al., 2006). These models accelerated our understanding of the circuitry and neurobiological underpinnings of SUDs, providing intriguing new pharmacotherapeutic targets for OUD and CUD. One such target is ghrelin, known as the “hunger hormone.” Ghrelin binds to the growth hormone secretagogue receptor 1α (GHS1αR) to play crucial roles in appetite and adiposity, as well as modulatory roles in many organ systems, including the brain (for reviews) (Engel and Jerlhag, 2014; Zallar et al., 2017). The GHS1αR is a G protein-coupled receptor with high constitutive activity and complex pharmacology (Ramirez et al., 2019). Small molecules with selectivity as GHS1αR antagonists and inverse agonists have been developed for therapeutic applications, including metabolic diseases and obesity (for reviews) (Cameron et al., 2014; Kumar, 2019; Ramirez et al., 2019), including the peptidergic GHS1αR full antagonist JMV2959 (Demange et al., 2007; Moulin et al., 2007; Ramirez et al., 2019).

Converging literature suggests that central ghrelin actions influence the neurochemical and behavioral effects of alcohol, cocaine, and opioid agonists (Jang et al., 2013; Schuette et al., 2013; Sustkova-Fiserova et al., 2017; Abtahi et al., 2018; Zallar et al., 2018; You et al., 2022a; You et al., 2022b; Richardson et al., 2023). While ghrelin administered centrally or systemically increases alcohol consumption (Jerlhag et al., 2009; Farokhnia et al., 2018a), these outcomes are blocked in rodents treated with the brain-penetrant GHS1αR antagonist JMV2959 or other GHS1αR antagonists (Jerlhag et al., 2009; Landgren et al., 2012; Suchankova et al., 2013; Gomez et al., 2015). In human psychopharmacology studies, ghrelin increases both alcohol self-administration and alcohol-seeking in heavy alcohol drinkers, while the clinically available GHS1αR inverse agonist PF5190457 suppresses these behaviors in a similar population (Leggio et al., 2014; Farokhnia et al., 2018b; Lee et al., 2018). Ghrelin potentiates (Dunn et al., 2019) and JMV2959 suppresses (Jerlhag et al., 2010; Wenthur et al., 2019; Sustkova-Fiserova et al., 2020) cocaine conditioned place preference (CPP), an assay that taps into the rewarding effects of drug-associated contextual cues (Bardo and Bevins, 2000; Huston et al., 2013). Intriguingly, lever presses for cocaine-associated cues positively correlates with ghrelin levels in rats trained in cocaine self-administration (Tessari et al., 2007). Intraventricular ghrelin infusion also increases heroin self-administration (Maric et al., 2012), although infusion of the GHS1αR antagonist [D-Lys-3]-GHRP-6 does not alter heroin intake or reinstatement of heroin-seeking evoked by food deprivation (Maric et al., 2012). Systemic administration of JMV2959 decreases expression of morphine CPP in rodents (Jerlhag et al., 2010; Engel et al., 2015; Jerabek et al., 2017), and blunts fentanyl self-administration in rats (Sustkova-Fiserova et al., 2020). Thus, a GHS1αR antagonist may be of utility in mitigating aspects of SUDs which sustain drug intake or promote relapse vulnerability during abstinence.

The present preclinical studies were designed to investigate the relative effectiveness of JMV2959 to impact cocaine or opioid self-administration and drug-seeking. While premised by previous cocaine and opioid self-administration findings, we analyze JMV2959 in a rat self-administration assay in a different strain (Sprague-Dawley) and under distinct operant conditions than prior publications (Sustkova-Fiserova et al., 2020; You et al., 2022a; You et al., 2022b). We selected oxycodone as a highly misused opioid in humans which readily supports self-administration in rodents (Pravetoni et al., 2014; Wade et al., 2015; Neelakantan et al., 2017), has a fast onset of action likely related to its high cerebral accumulation and active influx transport through the blood-brain barrier and a long duration of action (Olkkola et al., 2013). We analyzed the effectiveness of JMV2959 to suppress cocaine or oxycodone self-administration and drug-seeking within a dose-range of JMV2959 (0.5–2 mg/kg) that lacks impact on general behavioral parameters (Landgren et al., 2012; Suchankova et al., 2013; Sustkova-Fiserova et al., 2016; Jerabek et al., 2017; Rodriguez et al., 2018). Thus, in the present study, we tested the hypothesis that pretreatment with the GHS1αR antagonist JMV2959 (0.5–2 mg/kg) would suppress cocaine and/or oxycodone self-administration and cue-induced drug-seeking in a dose-related manner.

## Materials and methods

### Animals

Naïve, male Sprague-Dawley rats (*n* = 58, Harlan, Inc., Indianapolis, IN, USA) weighing 250–300 g upon arrival were acclimated to their home cages for 7 days. Rats were pair-housed in the animal colony on a 12-hour light-dark cycle, with temperature (21°C–23°C) and humidity (45%–50%) held constant. Rats had *ad libitum* access to water and standard chow (Teklad LM-485 Mouse/Rat Sterilizable Diet; Teklad Diets, Madison, WI, USA) throughout the experiments, except during daily self-administration sessions and cue-reinforced drug-seeking tests. All experiments were conducted during the light cycle and were approved by the University of Texas Medical Branch Institutional Animal Care and Use Committee and followed guidelines set forth by the NIH *Guide for the Care and Use of Laboratory Animals* (2011).

### Drugs

(-)-Cocaine hydrochloride (National Institutes on Drug Abuse, Research Triangle Park, NC, USA), JMV2959 (MilliporeSigma, Burlington, MA, USA), and oxycodone hydrochloride (Sigma, Research Triangle Park, NC, USA) were each dissolved in saline (0.9% NaCl) which served as the vehicle for all studies.

### Indwelling jugular catheter surgery

Rats were anesthetized with a cocktail of xylazine (8.6 mg/kg), acepromazine (1.5 mg/kg), and ketamine (43 mg/kg), and indwelling catheters were implanted into the right jugular vein. Catheters were secured to a plastic back mount and surgical mesh, as previously described (Cunningham et al., 2011; Neelakantan et al., 2017; Sholler et al., 2019), and rats were allowed 7 days of recovery after surgery. Catheter patency was maintained with a bacteriostatic saline infusion (0.1 mL) containing streptokinase (0.67 mg/mL; Sigma Chemical, St. Louis, MO, USA), heparin sodium (10 U/mL; American Pharmaceutical Partners, East Schaumburg, IL, USA), and ticarcillin disodium (66.67 mg/mL; Research Products International, Mt. Prospect, IL, USA) administered immediately after daily self-administration sessions.

### Apparatus

All self-administration sessions were conducted in standard operant chambers containing two retractable levers, cue lights above each lever, and a house light on the opposite side of the chamber (Med-Associates, Inc., St. Albans, VT, USA). The operant chambers were contained in sound-attenuated, ventilated boxes (Med-Associates, Inc.). Intravenous infusions of cocaine or oxycodone were delivered via syringes attached to infusion pumps (Med-Associates, Inc.) with polyethylene 20 tubing. The polyethylene tubing inside the operant chamber was encased and protected with a metal spring leash (Plastics One, Roanoke, VA, USA) secured to a swivel (Instech, Plymouth Meeting, PA, USA) that allowed unobstructed access to the entire operant chamber.

## Experimental design and procedures

### Effects of GHS1αR blockade on cocaine intake and cue-reinforced cocaine-seeking

Freely fed rats (*n* = 8) were trained to self-administer cocaine (0.25 mg/kg/infusion) in daily 60-min sessions (Cunningham et al., 2011) which results in stability at ∼15 infusions/session. This training dose lies near the peak of the cocaine self-administration dose-response curve and thus provides a large window of behavior that may be altered by pharmacological intervention (Piazza et al., 2000). Active lever presses were reinforced with a cocaine infusion and simultaneous presentation of the discrete cue complex (house light, cue light, and sound of infusion pump) for 6 seconds. The house light remained illuminated for 20 s to signal a time out interval during which presses on the active lever were counted but had no consequences. Throughout the self-administration sessions, inactive lever presses had no scheduled consequences. Rats acquired cocaine self-administration on a fixed ratio 1 (FR1) schedule of reinforcement and advanced to a FR5 schedule until meeting the stability criteria (<10% variability in infusions earned between sessions for at least three consecutive sessions). During acquisition, one rat lost full catheter patency, and was excluded from subsequent analyses (*n* = 7). In a within-subjects design, rats were treated with vehicle (saline; 1 mL/kg) or JMV2959 (0.5, 1, or 2 mg/kg; i.p.) 20 min prior to a cocaine self-administration session; the order of drug pretreatment was randomized across the study. Each test day was separated by a minimum of two intervening sessions to ensure rats exhibited stable self-administration behavior between challenges.

A separate cohort of freely fed rats (*n* = 18) was trained on cocaine self-administration (0.75 mg/kg/infusion) in daily 180-min sessions using the same stability criteria described above; this paradigm results in robust cue-reinforced cocaine-seeking during abstinence (Cunningham et al., 2013; Anastasio et al., 2014; Swinford-Jackson et al., 2016; Miller et al., 2018; Sholler et al., 2019). One rat lost full catheter patency and was excluded from subsequent analyses (*n* = 17). Following acquisition of stable cocaine self-administration on a FR5 schedule, rats were returned to their home cages for 24 h after which rats were treated with either vehicle (saline; 1 mL/kg) or JMV2959 (1 or 2 mg/kg; i.p.) 20 min prior to initiation of the cocaine-seeking test. Rats were pseudo-randomly assigned to three treatment groups (*n* = 5–6 rats/treatment) to assure that total lifetime cocaine intake (infusions) were similar. During the 60-min test session, presses on the previously active lever resulted in delivery of the discrete cue complex on an FR1 schedule, but no cocaine infusion. Presses on the inactive lever had no scheduled consequences.

### Effects of GHS1αR blockade on oxycodone intake and cue-reinforced oxycodone-seeking

Freely fed rats (*n* = 14) were trained to self-administer oxycodone (0.1 mg/kg/infusion) in daily 180-min sessions (Beardsley et al., 2004; Pravetoni et al., 2014; Neelakantan et al., 2017). The oxycodone self-administration task parameters were identical to the cocaine self-administration parameters described above. Briefly, rats acquired self-administration on a FR1 schedule of reinforcement and advanced to an FR3, then an FR5 schedule once stability criteria (above) had been met. Four rats either lost catheter patency or did not meet stability criteria prior to the start of pharmacological testing and were excluded from subsequent analyses (*n* = 10 rats). In a within-subjects design, vehicle or JMV2959 (0.5, 1, or 2 mg/kg; i.p.) was tested with at least 2 days of stable oxycodone self-administration between tests; the order of pretreatment was randomized across the study.

A separate cohort of freely fed rats (*n* = 18) was trained to self-administer oxycodone (0.1 mg/kg/infusion) as described above (Neelakantan et al., 2017). The cue-reinforced oxycodone-seeking test was conducted 24 h after the last self-administration session. Rats were treated with vehicle or JMV2959 (1 or 2 mg/kg; i.p.) 20 min prior to initiation of the test. Rats were pseudo-randomly assigned to treatment groups (*n* = 6 rats/treatment) to assure that total lifetime oxycodone infusions did not differ between treatment groups. Previously active lever presses were reinforced with the discrete cue complex on an FR1 schedule, but oxycodone was not infused. Inactive lever presses had no scheduled consequences.

### Statistical analyses

The self-administration data (infusions earned, lever presses, latency to first response) during self-administration maintenance and drug-taking test sessions were analyzed using a within-subjects one-way analysis of variance (ANOVA) (GraphPad Prism version 9.0.0). Previously active lever presses, inactive lever presses, and latency to first response during cue-reinforced drug-seeking tests were analyzed separately with a between-subjects one-way ANOVA. Planned comparisons with Dunnett’s procedure were assessed only when overall significance for the main effect of treatment was met for all ANOVAs. All statistical analyses were conducted with an experiment-wise error rate of α = 0.05. Effect sizes (Cohen’s d) were calculated using G*Power (Version 3.1.9.4). Researchers who administered compounds and conducted endpoint analyses were blinded to group assignments.

## Results

### JMV2959 does not suppress cocaine intake

**FIGURE 1:**
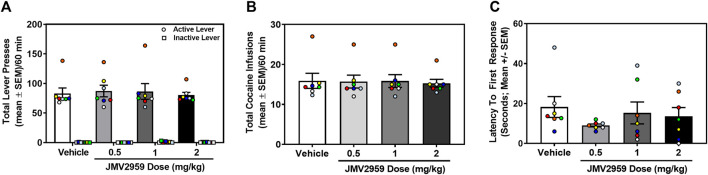
JMV2959 does not alter cocaine intake (within-subjects, *n* = 7). **(A)** JMV2959 (0.5, 1, 2 mg/kg) does not reduce the total number of active (mean ± SEM/60 min) or inactive lever presses (mean ± SEM/60 min). **(B)** JMV2959 (0.5, 1, 2 mg/kg) failed to reduce the total number of cocaine infusions (mean ± SEM/60 min) relative to vehicle. **(C)** Latency to the first response is not significantly altered by JMV2959. All datapoints represent *n* = 7 rats (denoted by circles/squares) in a within-subjects design.

Rats acquired stable cocaine self-administration (0.25 mg/kg/infusion) (data not shown). Across the last three sessions, there was no main effect of session on the number of infusions earned (daily mean ± SEM = 16.14 ± 2.72, 16.71 ± 2.33, 16.00 ± 2.40) (F_2,12_ = 1.64, *p* = 0.24), active (daily mean ± SEM = 86.43 ± 13.26, 92.57 ± 14.86, 89.00 ± 17.20) (F_2,12_ = 0.99, *p* = 0.40), or inactive lever presses (daily mean ± SEM = 0.29 ± 0.18, 0.14 ± 0.14, 0.14 ± 0.014) (F_2,12_ = 0.22, *p* = 0.80). A within-subjects one-way ANOVA revealed that JMV2959 (0.5, 1, 2 mg/kg; i.p.) did not alter cocaine infusions (F_3,18_ = 0.36, *p* = 0.78; Figure 1A), active lever presses (F_3,18_ = 0.45, *p* = 0.72; Figure 1B) or inactive lever presses (F_3,18_ = 2.23, *p* = 0.12; Figure 1B), relative to vehicle treatment. Latency to the first lever response was also unaltered by JMV2959 treatment (F_3,18_ = 1.41, *p* = 0.28; Figure 1C). Thus, JVM2959 (0.5, 1, 2 mg/kg) did not alter cocaine intake or other measures in this assay at the current training dose (0.25 mg/kg/infusion).

### JMV2959 suppresses cue-reinforced cocaine-seeking

**FIGURE 2:**
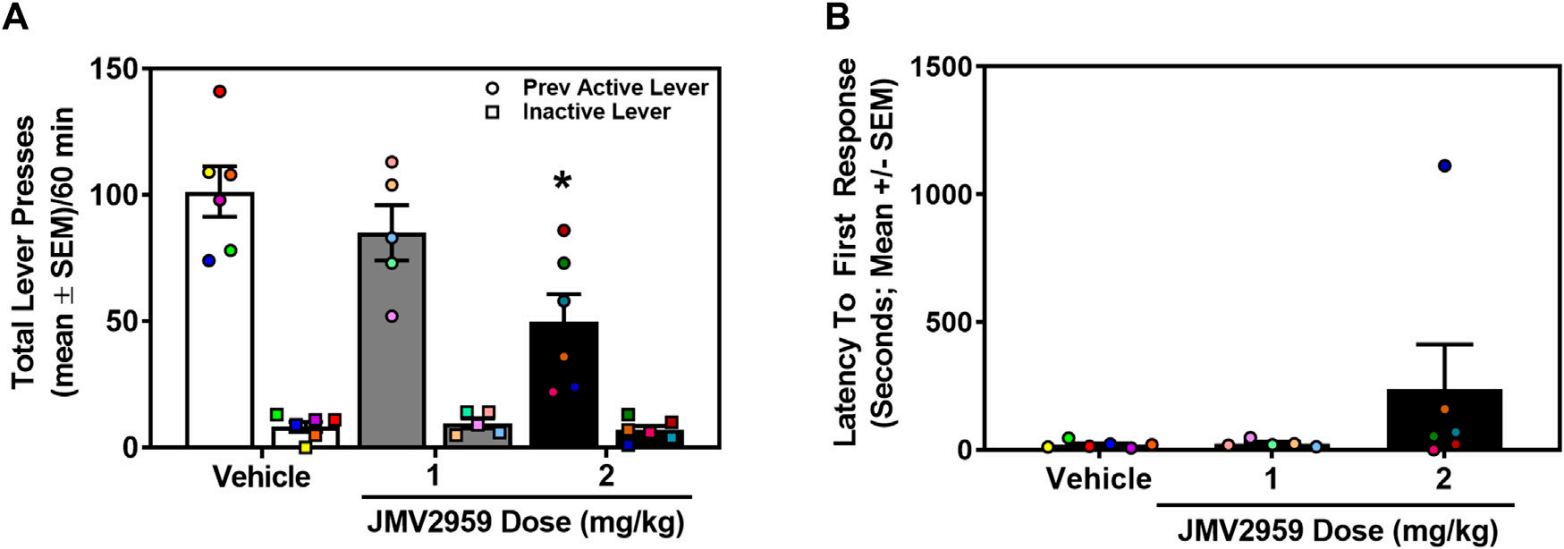
JMV2959 suppresses cue-reinforced cocaine-seeking (between-subjects, *n* = 5–6/treatment). **(A)** JMV2959 (2 mg/kg) decreases total number of previously active (circles), but not inactive lever presses (squares) assessed in the 60-min test session (mean ± SEM/60 min). **(B)** Latency to the first response is not significantly altered by JMV2959. *p < 0.05 vs. vehicle. All datapoints represent *n* = 17 rats (denoted by circles/squares) in a between-subjects design.

A separate cohort of rats acquired stable cocaine self-administration (0.75 mg/kg/infusion) (data not shown). Across the last three sessions of cocaine self-administration, there was no main effect of session on the number of infusions earned (daily mean ± SEM = 41.59 ± 1.08, 40.59 ± 0.97, 40.82 ± 1.15) (F_2,32_ = 1.07, *p* = 0.35), active (daily mean ± SEM = 232.76 ± 9.53, 228.71 ± 8.48, 224.19 ± 7.90) (F_2,32_ = 1.27, *p* = 0.29), or inactive lever presses (daily mean ± SEM = 1.00 ± 0.39, 0.76 ± 0.30, 0.47 ± 0.17) (F_2,32_ = 0.84, *p* = 0.44). The total number of lifetime cocaine infusions earned (mean ± SEM) prior to cocaine-seeking assessment did not differ in rats assigned to vehicle (458.0 ± 30.80 infusions), 1 mg/kg (476.80 ± 41.25 infusions) or 2 mg/kg (460.67 ± 38.46 infusions) treatment (F_2,14_ = 0.07, *p* = 0.93). Therefore, prior to test, each treatment group exhibited a comparable cocaine self-administration history. During the cue-reinforced cocaine-seeking session, a between-subjects one-way ANOVA revealed a main effect of treatment on previously active lever presses (F_2,14_ = 6.50, *p* = 0.01; Figure 2A). Dunnett’s planned comparisons indicated that the 2 mg/kg dose of JMV2959 significantly reduced previously active lever presses (*p* = 0.01, effect size d = 2.02), while the 1 mg/kg dose of JMV2959 had no effect (*p* = 0.47) when compared to vehicle. There was no main effect of treatment on inactive lever presses (F_2,14_ = 0.52, *p* = 0.61; Figure 2A) or latency to first response (F_2,14_ = 1.32, *p* = 0.30; Figure 2B). Thus, although JMV2959 did not alter cocaine intake (Figure 1A), 2 mg/kg of JVM2959 suppressed cue-reinforced cocaine-seeking.

### JMV2959 does not suppress oxycodone intake

**FIGURE 3:**
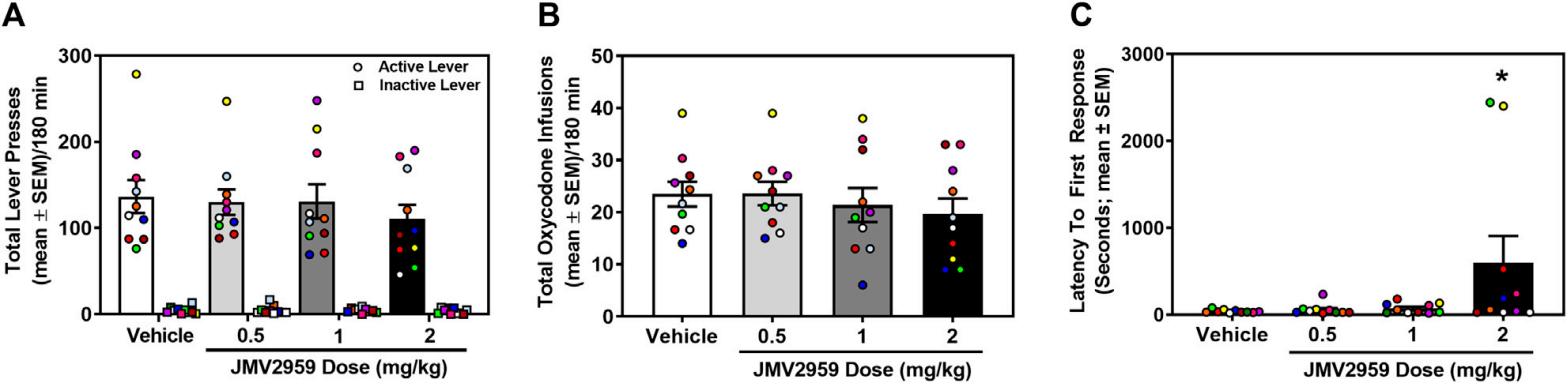
JMV2959 does not alter oxycodone intake (within-subjects, *n* = 10). **(A)** The total number of active (circles; mean ± SEM/180 min) and not inactive lever presses (squares; mean ± SEM/180 min) are presented. **(B)** JMV2959 (0.5, 1, 2 mg/kg) does not impact the total number of oxycodone infusions (mean ± SEM/180 min) relative to vehicle. **(C)** Latency to the first response is significantly increased by the highest dose of JMV2959 (2 mg/kg). *p < 0.05 vs. vehicle. All datapoints represent *n* = 10 rats (denoted by circles/squares) in a within-subjects design.

Rats acquired stable oxycodone self-administration (0.1 mg/kg/infusion) (data not shown). Across the last three sessions of oxycodone self-administration, there was no main effect of session on the number of infusions earned (daily mean ± SEM = 23.00 ± 2.53, 23.30 ± 2.84, 23.50 ± 2.55) (F_2,18_ = 0.40, *p* = 0.68), active (daily mean ± SEM = 120.10 ± 14.74, 126.80 ± 16.52, 128.50 ± 15.01) (F_2,18_ = 1.50, *p* = 0.25), or inactive lever presses (daily mean ± SEM = 4.00 ± 1.37, 5.20 ± 1.51, 4.30 ± 1.16) (F_2,18_ = 0.36 *p* = 0.70). A within-subjects one-way ANOVA revealed no significant treatment effect on oxycodone infusions earned (F_3,27_ = 1.17, *p* = 0.34; Figure 3A) active lever presses (F_3,27_ = 0.90, *p* = 0.45; Figure 3B) or inactive lever presses (F_3,27_ = 0.42, *p* = 0.74; Figure 3B). However, latency to first response was significantly impacted by JMV2959 (F_3,27_ = 3.14, *p* = 0.04; Figure 3C) with the highest dose (2 mg/kg) significantly increasing latency, relative to vehicle treatment (*p* = 0.04). Thus, JMV2959 (0.5, 1, 2 mg/kg) did not impact oxycodone intake at the current training dose (0.1 mg/kg/infusion).

### JMV2959 suppresses cue-reinforced oxycodone-seeking

**FIGURE 4:**
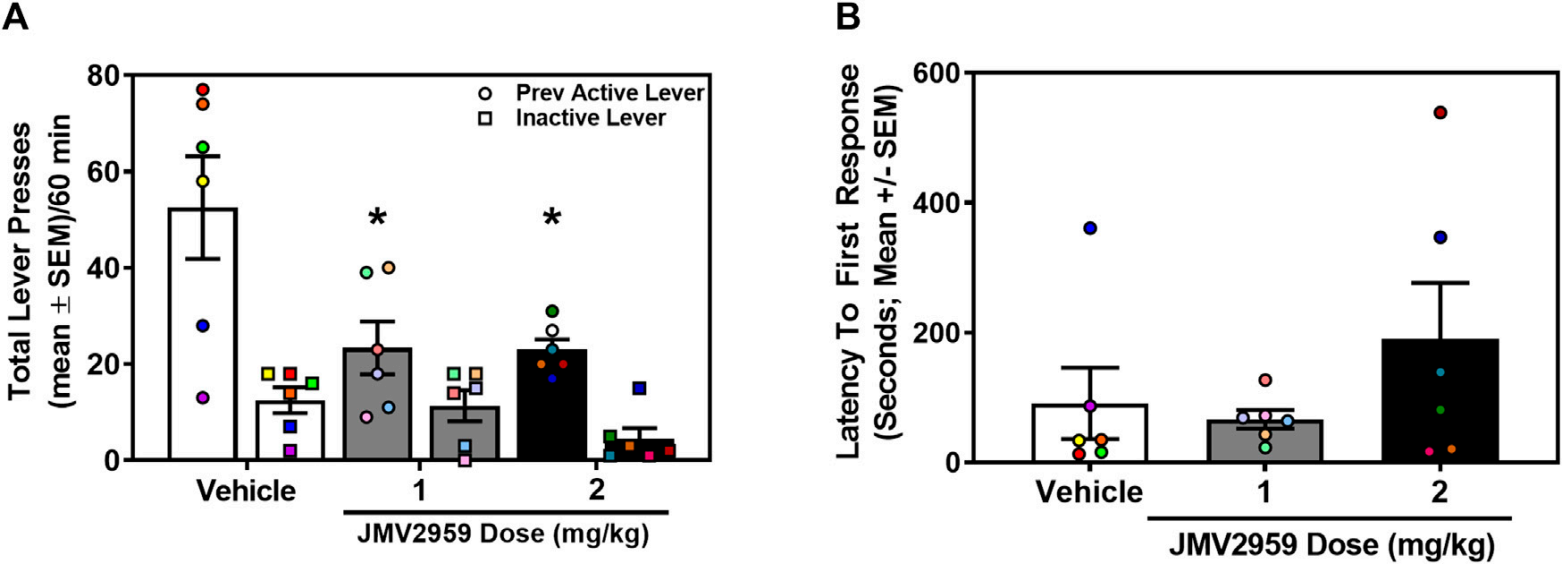
JMV2959 suppresses cue-reinforced oxycodone-seeking (between-subjects, n = 6/treatment). **(A)** JMV2959 (1 and 2 mg/kg) decreases total number of previously active lever presses (circles), but not inactive lever presses (squares) assessed in the 60-min test session (mean ± SEM/60 min). **(B)** Latency to the first response is not significantly altered by JMV2959. All datapoints represent *n* = 18 rats (denoted by circles/squares) in a between-subjects design. *p < 0.05 vs. vehicle.

A separate cohort of rats acquired stable oxycodone self-administration (data not shown). Across the last three sessions of oxycodone self-administration, there was no main effect of session on the number of infusions earned (daily mean ± SEM = 26.61 ± 2.69, 27.00 ± 2.47, 26.67 ± 2.37) (F_2,34_ = 2.04, *p* = 0.15), active (daily mean ± SEM = 164.00 ± 25.98, 162.06 ± 22.67, 152.28 ± 21.93) (F_2,34_ = 3.17, *p* = 0.06), or inactive lever presses (daily mean ± SEM = 3.11 ± 0.69, 4.28 ± 1.01, 2.44 ± 0.54) (F_2,34_ = 1.53, *p* = 0.23). The total number of lifetime oxycodone infusions earned (mean ± SEM) prior to oxycodone-seeking assessment did not differ in rats assigned to vehicle (344.17 ± 45.96 infusions), 1 mg/kg (360.50 ± 50.25 infusions) or 2 mg/kg (351.83 ± 51.99 infusions) treatment (F_2,15_ = 0.03, *p* = 0.97). Therefore, prior to the oxycodone-seeking test, all treatment groups exhibited a comparable oxycodone self-administration history. The between-subjects one-way ANOVA revealed a main effect of treatment on previously active lever presses (F_2,15_ = 5.80, *p* = 0.01; Figure 4A). Dunnett’s planned comparisons indicated that 1 mg/kg (*p* = 0.02, effect size d = 1.40) and 2 mg/kg (*p* = 0.02, effect size d = 1.57) of JMV2959 suppressed previously active lever presses when compared to vehicle. There was no main effect of treatment on inactive lever presses (F_2,15_ = 2.52, *p* = 0.11; Figure 4A) or latency to first response (F_2,15_ = 1.23, *p* = 0.32; Figure 4B). Thus, JMV2959 suppressed cue-evoked oxycodone-seeking.

## Discussion

Accumulated findings suggest that JMV2959 is efficacious in suppressing the *in vivo* neurochemical and behavioral effects induced by cocaine and opioid agonists, premising the studies conducted here. We found that under the current experimental conditions, JMV2959 does not impact self-administration of cocaine nor oxycodone. This is in direct contrast with previous studies that show pharmacological blockade of the GHS1αR via JMV2959 is sufficient to blunt cocaine and oxycodone intake (You et al., 2022a; You et al., 2022b). It is possible that the doses of JMV2959 employed here (0.5–2 mg/kg) were below the effective threshold to supersede the reinforcing properties of either self-administration training drug; JMV2959 was reported to blunt oxycodone intake when administered at doses of 2.5 mg/kg or higher (You et al., 2022b). Several additional experimental factors may also explain the discrepancies between the current and published studies (e.g., rat strain, dose of training drug, session length, schedule of reinforcement). Conversely, JMV2959 robustly decreased responding for the discrete cue complex previously associated with either cocaine or oxycodone delivery in male rats in the present study. In agreement, JMV2959 dose-dependently reduced cocaine- and stress-primed reinstatement of cocaine-seeking in male Long-Evans rats (You et al., 2022a). The dose range of JMV2959 (0.5–2 mg/kg) employed here did not alter operant responses on the inactive lever, but increased latency to the first response in the oxycodone-taking study. This effect is primarily driven by two of the rats assessed (*n* = 10) (Figure 3C). Doses of JMV2959 exceeding 3 mg/kg have been noted to alter general behavioral measures in previous studies in male Wistar or Long-Evans rats and thus were avoided (Landgren et al., 2012; Sustkova-Fiserova et al., 2014; Sustkova-Fiserova et al., 2016; Jerabek et al., 2017). Importantly, our unpublished data indicate that JMV2959 treatment does not alter locomotor activity under the same conditions employed in the present studies (dose, route of administration, and pretreatment time). Male Sprague-Dawley rats received JMV2959 (0–2 mg/kg; i.p.; 20 min) and were placed into open field chambers for 2 hours. A between-subjects two-way ANOVA with the factors of time and treatment time reveal a main effect of time (F_23,644_ = 92.87, *p* < 0.01), but no main effect of treatment (F_3,28_ = 0.63, *p* = 0.60) or time × treatment interaction (F_69,644_ = 0.92, *p* = 0.69). Taken together, these experiments provide compelling evidence that the GHS1αR-ghrelin axis regulates aspects of relapse-like events associated with both cocaine and oxycodone.

Ghrelin is proposed to cross the blood-brain barrier or affect vagal function to evoke centrally mediated effects ultimately through modulation of the meso-corticolimbic, hypothalamic, and hippocampal pathways (Banks et al., 2002; Zigman et al., 2006; Cabral et al., 2017; Rhea et al., 2018). Of relevance to SUDs, the mesolimbic dopamine (DA) pathway from ventral tegmental area (VTA) DA cell bodies to the nucleus accumbens (NAc) is a chief mediator of the rewarding properties of misused drugs and drug-associated cues (for review) (Volkow et al., 2016). The VTA is a key region in which ghrelin is thought to promote motivation for hedonic rewards (for review) (Abizaid et al., 2006). The GHS1αR transcript is enriched in the VTA (Guan et al., 1997; Kojima et al., 1999) and, although expressed in both VTA DA and γ-aminobutyric acid (GABA) neurons (Abizaid et al., 2006; Zigman et al., 2006), accumulated data suggest that the actions of ghrelin in the VTA are mediated by GHS1αR-expressing DA, but not GABA, neurons (Abizaid et al., 2006). Systemic injection of JMV2959 reduces both cocaine- (Jerlhag et al., 2010) and morphine-induced DA efflux in the NAc (Sustkova-Fiserova et al., 2014; Jerabek et al., 2017), presumably from DA neuronal terminals originating in the VTA. Moreover, acquisition of oxycodone self-administration elevates GHS1αR mRNA expression in VTA neurons containing the dopamine transporter (DAT), but not the vesicular GABA transporter (vGAT), further evidencing involvement of VTA dopaminergic cells in oxycodone-motivated behavior (You et al., 2022b). Based upon these findings, JMV2959 was expected to block the GHS1αR localized to VTA DA neurons, reducing the elevation in resulting NAc DA concentrations, and accordingly impacting both cocaine and oxycodone self-administration. However, JMV2959 failed to reduce the intake of either drug under the current experimental conditions.

The interpretation of these findings requires conjecture within the context of the target actions of cocaine and oxycodone to impact the mesoaccumbens DA pathway. The entirety of their pharmacological actions *in vivo* are not identical, however, both cocaine (Pettit et al., 1990; Weiss et al., 1992) and oxycodone (Vander Weele et al., 2014) result in overflow of NAc DA efflux. Cocaine has a high affinity for monoamine transporters, with the inhibition of DA reuptake processes playing a prominent role in its reinforcing effects (Ritz et al., 1987). In our hands, JMV2959 pretreatment did not alter self-administration at a cocaine dose (0.25 mg/kg/infusion) which is associated with sustained NAc DA overflow at ∼300% of baseline across the session (Pettit et al., 1990; Weiss et al., 1992). A dose of JMV2959 (6 mg/kg) considerably higher than those doses employed here (0.5–2 mg/kg) suppressed, but did not eliminate, NAc DA overflow evoked by an investigator-delivered dose of cocaine (10 mg/kg) (Jerlhag et al., 2010), suggesting that achieved levels of cocaine-evoked DA in the NAc may be insurmountable by the JMV2959 doses employed in the present study.

The functional role of the GHS1αR in neural nodes is tied to reward processes and drug-seeking behaviors (Jang et al., 2013; Schuette et al., 2013; Sustkova-Fiserova et al., 2017; Abtahi et al., 2018). Ghrelin is predominantly produced in the stomach mucosa (Kojima et al., 1999) and is proposed to cross the blood-brain barrier to evoke centrally mediated effects, although there is also evidence for expression of ghrelin-containing neurons within the brain (Banks et al., 2002; Hou et al., 2006; Rhea et al., 2018). Despite the uncertainty surrounding the source of ghrelin in the brain, converging literature suggests that central GHS1αR action or stimulation underlie the efficacy of GHS1αR ligands to influence the behavioral effects of misused drugs, and supports the premise that this system modulates behaviors with underlying deficits in prefrontal executive control, such as cue-evoked drug-seeking (Wellman et al., 2012; Jang et al., 2013; Schuette et al., 2013; Edwards and Abizaid, 2017; Sustkova-Fiserova et al., 2017; Abtahi et al., 2018; Wenthur et al., 2019). Further, *ex vivo* biochemical data show that acute treatment with JMV2959 decreases the metabolism of the monoamine serotonin within the amygdala, a region implicated in withdrawal and negative affect in the absence of misused substances (Vestlund et al., 2019). Intra-amygdala infusion of a serotonin 5-HT_2C_ receptor agonist suppresses cocaine-seeking during a cue-reinforced reinstatement test (Pockros-Burgess et al., 2014), as does systemic administration (Cunningham et al., 2011; Cunningham et al., 2013). It is possible that an underlying mechanism through which GHS1αR blockade suppresses cocaine- and oxycodone-seeking is due to JMV2959-induced increases in amygdala serotonin levels and activation of the prominent expression of the 5-HT_2C_R population in this region (Pompeiano et al., 1994; Clemett et al., 2000). As discussed above, additional findings also suggest the GHSR1α and central ghrelin actions have been shown to interact with serotonergic systems (Schellekens et al., 2015) as well as dopaminergic (Jerlhag et al., 2011; Jerlhag et al., 2012; Navarro et al., 2022) and cannabinergic systems (Kola et al., 2008).

Intra-VTA JMV2959 suppresses cue-evoked heroin-seeking in food-restricted, but not sated rats, potentially due in part to the elevated plasma ghrelin levels and the neuroplastic changes associated with chronic food restriction (D'Cunha et al., 2013). The rats in our study were freely fed, yet still demonstrated a robust effect of systemic JMV2959 to suppress cue-evoked drug-seeking. One possibility is that the effects of systemic JMV2959 to suppress drug-seeking behavior may be due to effects localized to the prefrontal cortex (PFC) which exerts top-down control over cue-induced dopaminergic signaling from the VTA to the NAc (Koob and Volkow, 2010). Specifically, PFC glutamatergic outputs to the striatum serve a regulatory role over crucial cue-induced DA release (for review) (Kalivas and Volkow, 2005). The GHS1αR is resident in the PFC, but localized function in this region has yet to be studied; thus, it is possible that GHS1αR control of cue-evoked drug-seeking may be due to its actions in the PFC (Guan et al., 1997). Absolute GHS1αR deletion via CRISPR/Cas9 technology has been shown to decrease alcohol-motivated behavior in rats (Zallar et al., 2019a; Zallar et al., 2019b); studies designed to assess the impact of overall GHS1αR knockout relative to localized GHS1αR gene silencing within the PFC on opioid- and cocaine-motivated behaviors will be valuable in the future.

The present studies were conducted in male rats, and given sex-specific distinctions in the ghrelin-GHS1αR in rats, differences in drug self-administration and cue-reinforced drug-seeking are projected (Lopez-Ferreras et al., 2017; Labarthe et al., 2021). Additionally, male Sprague-Dawley rats are more sensitive to the antinociceptive effects of morphine than female rats, and to a lesser degree to the major metabolite (oxymorphone) of oxycodone, while no sex differences were observed in this regard for fentanyl (Peckham and Traynor, 2006). Future comparative studies are needed to ascertain the extent to which the effectiveness of JMV2959 to suppress drug reward-related behaviors is dependent upon sex and sex hormones (Sell et al., 2000; Zhou et al., 2002; Hinds et al., 2023). In addition, we employed a daily, short-access model of drug self-administration to determine the impact of GHSR1α blockade on drug intake and drug-seeking. Notably, long-access (>6 h) cocaine self-administration paradigms produce escalation of drug intake over time with appreciable differences in drug consumption between male and female rats (Algallal et al., 2020). A recent study also reported significant sex differences in escalation of oxycodone intake (12h/session), however this was only the case for one of four heterogeneous strains tested (Doyle et al., 2023). Escalated drug intake in these preclinical models align with several criteria for SUDs outlined by the Diagnostic and Statistical Manual of Mental Disorders 5 including development of tolerance and greater intake with extended use (Hasin et al., 2013). It is important to determine if the suppressive effects of JMV2959 on drug-motivated behaviors persist in long-access self-administration models.

The mechanisms of action for cocaine and oxycodone are independent yet each drug is a powerful reinforcer with high abuse potential. We identified that the GHS1αR antagonist JMV2959 mitigates distinct aspects of cocaine- and oxycodone-associated behaviors in male rats. Future studies are needed to further clarify specific mechanisms for the regulatory control of the ghrelin-GHS1αR axis over cocaine and opioid intake and drug-seeking.

## Data Availability

The original contributions presented in the study are included in the article, further inquiries can be directed to the corresponding author.

## Abbreviations

ANOVA, analysis of variance; CPP, conditioned place preference; CUD, cocaine use disorder; DA, dopamine; GABA, γ-aminobutyric acid; GHS1αR, growth hormone secretagogue receptor 1α; FR, fixed ratio; NAc, nucleus accumbens; OUD, opioid use disorder; PFC, prefrontal cortex; SUD, substance use disorder; VTA, ventral tegmental area.
